# Suppression of OSCC malignancy by oral glands derived‐PIP identified by iTRAQ combined with 2D LC‐MS/MS

**DOI:** 10.1002/jcp.28180

**Published:** 2019-01-28

**Authors:** Qibo Wang, Yuan Zhi, Wenhao Ren, Shaoming Li, Zhichao Dou, Xiaoming Xing, Xinyu Quan, Yuting Wang, Chunmiao Jiang, Xiao Liang, Ling Gao, Keqian Zhi

**Affiliations:** ^1^ Department of Oral Maxillofacial Surgery Key Lab of Oral Clinical Medicine, The Affiliated Hospital of Qingdao University Qingdao Shandong China; ^2^ School of Stomatology, Qingdao University Qingdao Shandong China; ^3^ Xiangya School of Stomatology, Central South University Changsha Hunan China; ^4^ Department of Research The Affiliated Hospital of Qingdao University Qingdao Shandong China; ^5^ Department of Neurology Haukeland University Hospital Bergen Norway

**Keywords:** iTRAQ, OSCC, PIP

## Abstract

Oral squamous cell carcinoma (OSCC) is the most common malignancy in head and neck cancer and a global cause of cancer‐related death. Due to the poor survival rates associated with OSCC, there is a growing need to develop novel technologies and predictive biomarkers to improve disease diagnosis. The identification of new cellular targets in OSCC tumors will benefit such developments. In this study, isobaric tags for relative and absolute quantitation (iTRAQ)‐based proteomics analysis combined with 2‐dimensional liquid chromatography and tandem mass spectrometry (2D LC‐MS/MS) were used to identify differentially expressed proteins (DEPs) between tumor and normal tissues. Of the DEPs detected, the most significantly downregulated protein in OSCC tissue was prolactin‐inducible protein (PIP). Clonogenic and 3‐(4,5‐dimethyl‐2‐thiazolyl)‐2,5‐diphenyl‐2H‐tetrazolium bromide (MTT) experiments showed that the proliferation capacity of OSCC cells overexpressing PIP decreased due to cell cycle arrest at the G0/G1 checkpoint. Wound‐healing and transwell assay further showed that PIP overexpression also reduced the migration and invasion of OSCC cells. Immunohistochemistry (IHC) was used to analyze the expression in OSCC, indicating that PIP may be secreted by glandular cells and have an inhibitory effect on OSCC cells to produce. In western blot analysis, silencing studies confirmed that PIP mediates these effects through the AKT/mitogen‐activated protein kinase (MAPK) signaling axis in OSCC cells. Taken together, this study reveals PIP as a key mediator of OSCC cell growth, migration, and invasion through its effect on AKT/MAPK signaling.

## 1 INTRODUCTION

Head and neck cancer is the sixth most common human malignancy worldwide and it is estimated that more than 90% of those are oral squamous cell carcinoma (OSCC; Choi & Myers, 2008; Markopoulos, 2012). Although remarkable improvements in OSCC therapy have occurred, including radiotherapy, chemotherapy, and surgical resection, the morbidity and mortality of OSCC have not significantly improved over the last decade, and the overall 5‐year survival rate for patients with OSCC remains low (Nakashima et al., 2017; Ono et al., 2018). The prognosis of OSCC is improved during early disease stages, particularly in well‐differentiated tumors that have not metastasized. However, the majority of patients with OSCC are diagnosed during the late stages of the disease. In this regard, new biomarkers are urgently required for early OSCC detection.

Isobaric tags for relative and absolute quantitation (iTRAQ) combined with 2‐dimensional liquid chromatography and tandem mass spectrometry (2D LC‐MS/MS) analysis have revealed promising candidate biomarkers for breast cancer, lung cancer, stomach cancer, colon cancer, and esophageal cancer. (Creaney, Dick, Leon, & Robinson, 2017; Sun et al., 2018; X. Wang et al., 2017, Y. Wang et al., 2017; Xiao et al., 2015; Yang, Chen, Chan, Li, & Zhang, 2017; Zhang et al., 2017). This technique permits the identification of differentially expressed proteins (DEPs) between tumor and normal tissues (Li et al., 2017). The traditional method for identifying DEPs is 2‐dimensional gel electrophoresis, but this fails to reliably identify DEPs of high molecular weight, or those are greatly acidic or basic or dwell in the cell membrane (Jiang et al., 2017).

Using the iTRAQ approach, an 8‐plex set of amine‐reactive isobaric tags are used to derivatize peptides at the N‐terminus and the lysine side chains for labeling all peptides (Chai et al., 2016). iTRAQ frequently yields highly quantitative data and has the advantage of high throughput achieved by sample multiplexing, high confidence of identification, and highly sensitive quantification of protein expression (Narayana, Tomatis, Wang, Kvaskoff, & Meunier, 2015; Negroni et al., 2014).

In this study, iTRAQ labeling was coupled with 2D LC‐MS/MS to detect the DEPs between OSCC and normal oral tissue (Xiao et al., 2015). Certain DEPs for binding were further selected and validated for their molecular function by Gene Ontology (GO) analyses. The value of ten DEPs (alpha‐actinin‐1 [ACTN1], apolipoprotein B mRNA editing enzyme cytidine deaminase [APOBEC3G], calsequestrin‐1 [CASQ1], dihydropyrimidinase‐related protein 2 [DPYSL2], filaggrin [FLG], filaggrin‐2 [FLG2], prolactin‐inducible protein [PIP], syntenin‐2 [SDCBP2], 45 kDa calcium‐binding protein [SDF4], and tropomyosin 1 [TPM1]) were assessed. The inhibitory effects of PIP in OSCC and adjacent normal tissues were further characterized (Tomar, Sooch, Raj, Singh, & Yadav, 2013). We found that PIP could regulate the proliferation, migration, and invasion of OSCC through AKT/mitogen‐activated protein kinase (MAPK) signaling. Using immunohistochemistry (IHC), we discovered that PIP is expressed in both gland and stromal cells, and it has been reported that PIP is related to secretory function. We therefore predict that PIP is secreted by acinar cells into the stroma to act on OSCC cells.

## 2 MATERIALS AND METHODS

### 2.1 Tissue samples and cell lines

Surgical tissue specimens were obtained from patients with OSCC diagnosed from 2016 to 2018 at the Affiliated Hospital of Qingdao University, China. Specimens were obtained from the surgical resection of OSCC primary tumors, along with paired samples of adjacent normal tissues. All samples were immediately frozen at −196°C (liquid nitrogen) and placed at −80°C for further storage. Ethical approval was ratified by the local Ethics Committee, and all patients provided written informed consent for the utilization of tissue samples in research.

The human OSCC cell lines, SCC15 and SCC25, were kindly provided by the Chinese Academy of Medical Sciences (Beijing, China). For the construction of the PIP overexpression plasmid, whole sequences of PIP gene were amplified from a human complementary DNA (cDNA) library, AsiSI and Mlu I restriction sites were inserted, and the construct cloned into a pEnter vector by Vigene Biosciences (Shandong, China). Small interfering RNAs for PIP (siRNA‐PIP) were designed by GenePharma (Shanghai, China). Primers were supplied by Thermo Fisher Scientific (Carlsbad, CA). The AKT inhibitor MK‐2206 and ERK inhibitor SCH772984 were obtained from ApexBio Technology (TX) and dissolved in dimethyl sulfoxide (DMSO) according to the manufacturer's instructions.

### 2.2 Isobaric tags for relative and absolute quantitation

Samples from liquid nitrogen were treated with 5 volumes of TCA (Trichloroacetic acid)/acetone (1:9), vortexed, and stored at −20°C for 4 hr. Samples were centrifuged at 6000*g* for 40 min at 4°C, supernatants were discarded and pellets washed in precooled acetone. The dried powder (20–30 mg) was weighed and 30 volumes (m/v) of SDT lysis buffer was added. Samples were vortexed and boiled in a water bath for 5 min. After centrifugation at 14000*g* for 15 min, supernatants were passed through a 0.22 μm filter and collected. Samples from OSCC tissues were labeled with 118, 119, and 121 reagents. Samples from matched normal tissues were labeled with 113, 114, and 115. Each group of labeled peptides were mixed and fractionated using an Agilent 1260 infinity II HPLC system (Agilent, Beijing, China).

### 2.3 Liquid chromatography and tandem mass spectrometry analyses

Samples were analyzed for 60 min using a Q‐Exactive mass spectrometer (Thermo Fisher Scientific, CA). The detection mode was the positive ion at a parent ion scan range of 350–1800 m/z and a primary mass spectrometer resolution of 70,000. The level one maximum IT was 50 ms. The mass‐to‐charge ratio of the peptides and polypeptide fragments were as follows: 10 MS spectra were acquired after each full scan. An HCD MS2 activation was used with an isolation window of 2 m/z. Secondary mass spectra were resolved at a rate was 17,500 (microscans = 1). The secondary maximum IT was 45 ms, and the normalized collision energy was 30 eV.

### 2.4 Bioinformatics analysis

Blast2GO annotation on the target protein sets was roughly divided into blast, mapping, annotation, and annotation augmentation. First, the NCBI BLAST + target protein alignment tool and appropriate protein sequence databases were used to meet the E‐value < = 1e−3 value. A total of 10 sequences were selected for subsequent analysis. Secondly, the Blast2GO Command Line was used to extract GO items associated with the qualified alignment sequences (database version: go_201504.obo, www.geneontology.org).

### 2.5 Quantitative real‐time PCR

Total RNA was isolated from surgical specimens or OSCC cell lines using a RNeasy Kit (TaKaRa, Dalian, China). cDNA was synthesized according to the manufacturer's instructions. SYBR Premix Ex Taq (TaKaRa) was used for real‐time polymerase chain reaction (RT‐PCR) at a total reaction volume of 15 μl. The cycling conditions included a holding step at 95°C for 30 s, followed by 40 cycles in two steps: 5 s at 95°C and 30 s at 60°C. A dissociation step was added to verify that each primer pair produced only a single product at 15 s at 95°C, 30 s at 55°C, and 15 s at 95°C. β‐Actin was used as an internal control. Gene expression was quantified using the $2^{{-\Delta \Delta C}_{t}}$ method.

### 2.6 Cell culture

Cells were cultured in high‐glucose Dulbecco's modified Eagle's Medium (DMEM) medium (HyClone, Logan, UT) supplemented with 10% fetal bovine serum (FBS; HyClone) at 37°C in a 5% CO_2_ standard humidified incubator. Cells in the logarithmic phase were used in all experiments. Cells were transfected using Lipofectamine 3000 (Thermo Fisher Scientific). Transfection efficiencies were validated through fluorescence microscopy (FSX100 Olympus, Tokyo, Japan).

### 2.7 Cell viability, cell cycle, and colony formation assay

For viability assays, cells were plated into 96‐well plates at a density of 5 × 10^4^ cells/ml for 24 hr and transfected. Cell viability was measured through MTT assays. For cell cycle analysis, cells were dissociated through trypsinization, fixed in 70% ethanol and stained with propidium iodide. Cell cycle distribution was analyzed by FlowJo 7.6.5 (Becton Dickinson, Franklin Lakes, NJ). For colony formation assays, 500 cells per well were seeded into six‐well plates and cultured for 12 days. Culture medium was replaced every 3 days. Viable colonies were scored through crystal violet staining. The average number of colonies was calculated and each experiment was repeated on three independent occasions.

### 2.8 Wound‐healing, cell migration, and invasion assays

For wound‐healing assays, control, PIP‐overexpressed, and PIP silenced OSCC cells were seeded into six‐well plates and grown to 100% confluency in complete medium. Linear scratches were made into cell monolayers using a pipette tip and cells were washed three times with phosphate‐buffered saline (PBS). Remaining cells were starved overnight in serum‐free medium to exclude the effects of proliferation on cell migration. Cell was imaged at 12 and 24 hr on a digital camera (Nikon, Kobe, Japan) and the scratch area was quantified using ImageJ 1.46r (National Institutes of Health, Bethesda, MA). Each experiment was repeated on three independent occasions.

For migration and invasion assays, transwell chambers (8‐μm pore size; Corning Costar, Dallas, TX) with or without Matrigel (100 μg/ml; BD Biosciences, San Jose, CA) were used to evaluate the migratory and invasion ability of SCC15 and SCC25 cells. For migration assays, cells were seeded into the upper chamber and cultured in serum‐free medium. The lower chamber was filled with medium containing 10% FBS. For invasion assays, Matrigel was precoated on the basement membrane of the upper chamber of the transwell. After 24 hr, five fields were randomly selected for counting on a light microscope (Nikon). Each experiment was performed on a minimum of three occasions and the average number of migrated or invaded cells calculated.

### 2.9 Immunohistochemistry

Formalin‐fixed paraffin‐embedded OSCC tissues and matched normal tissue (*n* = 45) were used for IHC studies. All samples were obtained from patients with informed consent and through approval of the Institutional Ethics Committee. IHC was performed using 1:100 anti‐PIP mouse affinity isolated (cat. no. ab218480; Abcam, Shanghai, China) primary antibodies. After deparaffinization and rehydration, antigen retrieval was performed by submerging the slides in antigen retrieval buffer (10 mM sodium citrate, pH 6.0) at 95°C for 20 min. Endogenous peroxidases were blocked for 10 min and slides were washed in 0.01 M PBS. Tissues were blocked in goat serum for 10 min and labeled with primary antibodies at 4°C overnight. After washing in 0.01 M PBS, 3,3′‐diaminobenzidine (DAB; Sangon Biotech, Shanghai, China) was added to visualize tissue antigens. Sections were counterstained with hematoxylin and dehydrated.

### 2.10 Protein–protein interaction network

The protein–protein interaction (PPI) network for PIP was retrieved from the Search tool for the retrieval of interacting proteins (STRING) database and reconstructed through the addition of recently identified PIP interacting proteins.

### 2.11 Western blot analysis

SCC15 and SCC25 cells were lysed in lysis buffer (Beyotime Biotechnology, Shanghai, China). Extracted proteins were separated by sodium dodecyl sulfate‐polyacrylamide gel electrophoresis and transferred onto polyvinylidene fluoride membranes (PVDF). Membrane was blocked in 5% nonfat dry milk in PBS‐Tween 20 (PBST) for 1 hr at room temperature and probed with primary antibodies against PIP, p‐AKT, AKT, p‐ERK (extracellular signal‐regulated kinase)1/2, ERK1/2, and β‐actin at 4°C overnight. After washing with PBST, membranes were labeled with the appropriate secondary antibodies for 1 hr at room temperature. Proteins were visualized using the enhanced chemiluminescence (Thermo Fisher Scientific, Rockford, IL).

### 2.12 Statistical analysis

All experiments were performed on a minimum of three occasions. Data are presented as the mean ± *SD* (standard deviation). Differences in the means of the test groups were compared through an analysis of variance (ANOVA) and *t* tests using the SPSS version 23.0 (Chicago, IL). Statistical significance was determined at *p* < 0.05 and *p* < 0.01.

## 3 RESULTS

### 3.1 Identification of DEPs between OSCC and normal samples by iTRAQ and LC‐MS/MS

**Figure 1:**
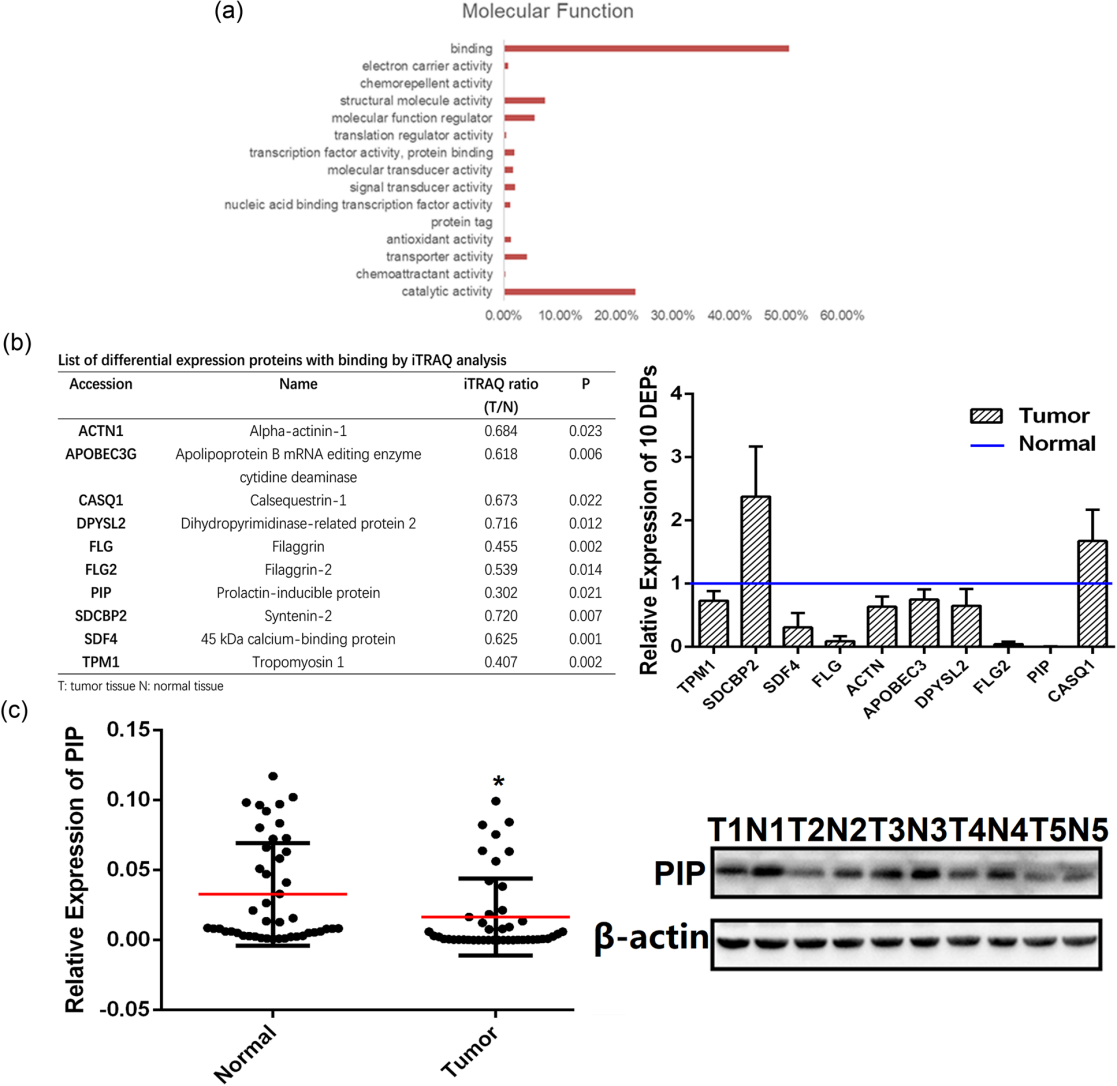
PIP detection in clinical specimens. (a) Molecular function of Gene Ontology (GO) analyses. (b) List of DEPs with binding by iTRAQ analysis; ten DEPs (ACTN1, APOBEC3G, CASQ1, DPYSL2, FLG, FLG2, PIP, SDCBP2, SDF4, TPM1) were assessed via qRT‐PCR analysis in OSCC and adjacent normal tissues (*n* = 29). (c) qRT‐PCR analysis of PIP expression in OSCC and adjacent normal tissues (*n* = 29); western blot analysis of PIP expression in OSCC and adjacent normal tissues (*n* = 5). The expression of PIP was normalized to β‐actin. Data were from three independent experiments and represented as mean ± *SD*. *p < 0.05. DEP: differentially expressed protein; iTRAQ: isobaric tags for relative and absolute quantitation; OSCC: oral squamous cell carcinoma; PIP: prolactin‐inducible protein; qRT‐PCR: quantitative real‐time polymerase chain reaction; *SD*: standard deviation [Color figure can be viewed at wileyonlinelibrary.com]

To identify potential OSCC biomarkers, we used iTRAQ combined with 2D LC‐MS/MS to analyze OSCC samples. Using the ProteinPilot software (AB Sciex, CA), a total of 1251 DEPs were identified, including 893 upregulated and 358 downregulated proteins. DEPs were classified according to GO terms including biological process, molecular function, and cellular compartment. For molecular function, the majority of proteins were involved in binding (50.52%), catalytic activity (23.30%), structural molecule activity (7.27%), molecular function regulator (5.44%), transporter activity (4.14%), and signal transducer activity (2.00%; Figure 1a). Ten proteins related to binding were randomly selected from the downregulated proteins, of which PIP was the most significant (*p* < 0.05; Figure 1b). We simultaneously selected 29 paired tissues for further verification by qPCR and five of those were validated by western blot analysis (Figure 1c).

### 3.2 PIP regulates cell proliferation and cell cycle progression

**Figure 2:**
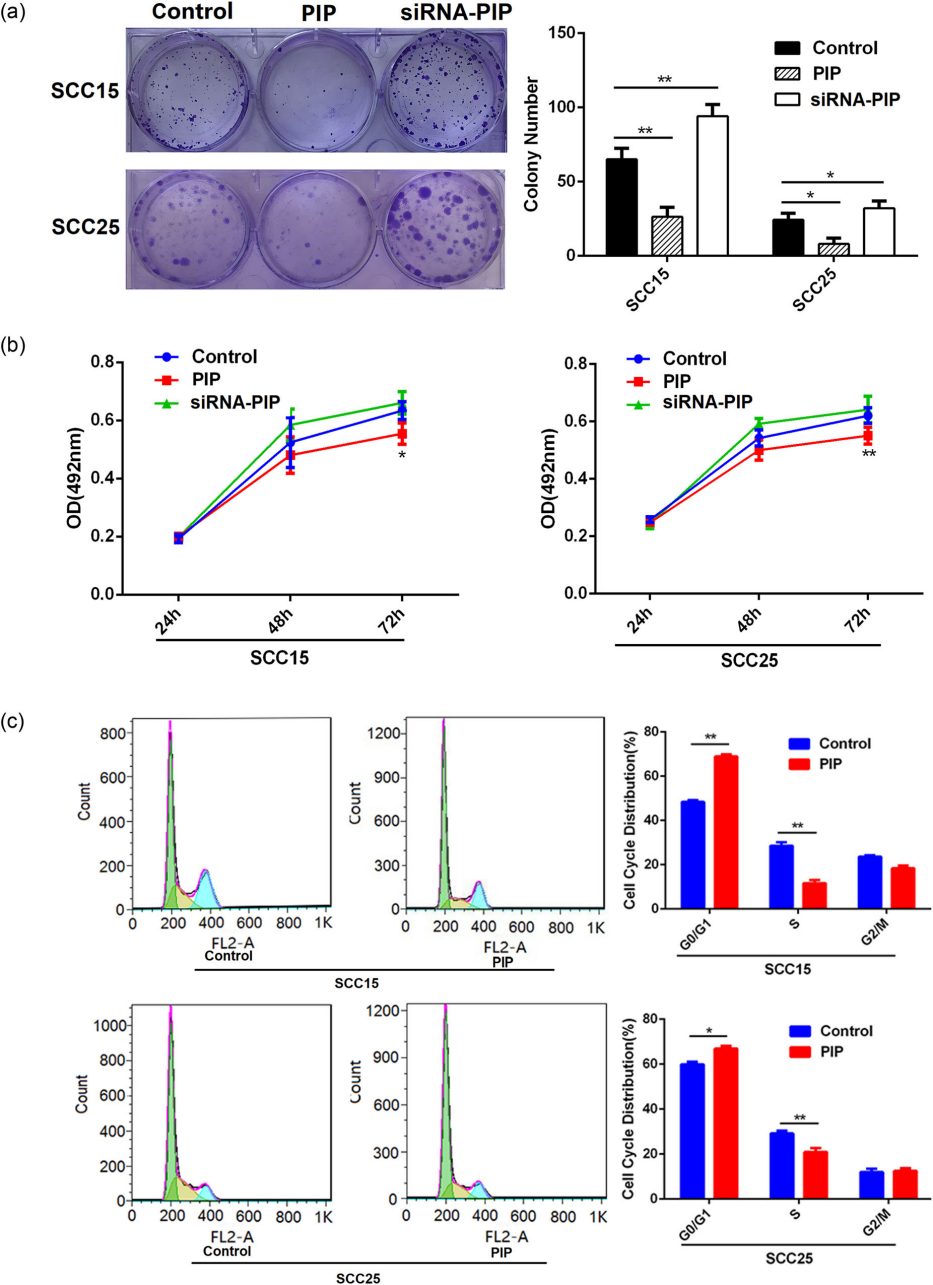
The growth effects of PIP on human OSCC cell lines. (a) After transfection with control vector, PIP vector or siRNA‐PIP, the image of 500 cells were severally plated in a six‐well plate for 12 days; the average colony number of per well. All data were shown as mean ± *SD*. *p < 0.05, **p < 0.01. (b) At 24, 48, and 72 hr after transfection with control, PIP vector or siRNA‐PIP, cell proliferation was examined by the MTT assay. All data were shown as mean ± *SD*. *p < 0.05, **p < 0.01. (c) The relative proportion of SCC15 and SCC25 cell cycle, after transfection with control vector and PIP vector. All data were shown as mean ± *SD*. *p < 0.05, **p < 0.01. MTT: 3‐(4,5‐Dimethyl‐2‐thiazolyl)‐2,5‐diphenyl‐2H‐tetrazolium bromide; OSCC: oral squamous cell carcinoma; PIP: prolactin‐inducible protein; *SD*: standard deviation; siRNA: small interfering RNA [Color figure can be viewed at wileyonlinelibrary.com]

The results from clonogenic assays revealed that the PIP overexpression in OSCC cells inversely correlates with control cells growth. Consistent with this finding, PIP silencing promoted colony numbers growth (*p* < 0.05; Figure 2a). MTT assays were used to examine the proliferation of PIP overexpressed cells and siRNA‐PIP cells versus control SCC15 and SCC25 cells. The cell proliferative capability of PIP overexpressed cells was significantly lower than control cells (*p* < 0.05; Figure 2b). Assessment of cell cycle status of PIP overexpressed cells revealed a G0/G1 phase arrest (*p* < 0.05; Figure 2c).

### 3.3 Effects of PIP on the migration and invasion of OSCC cells

**Figure 3:**
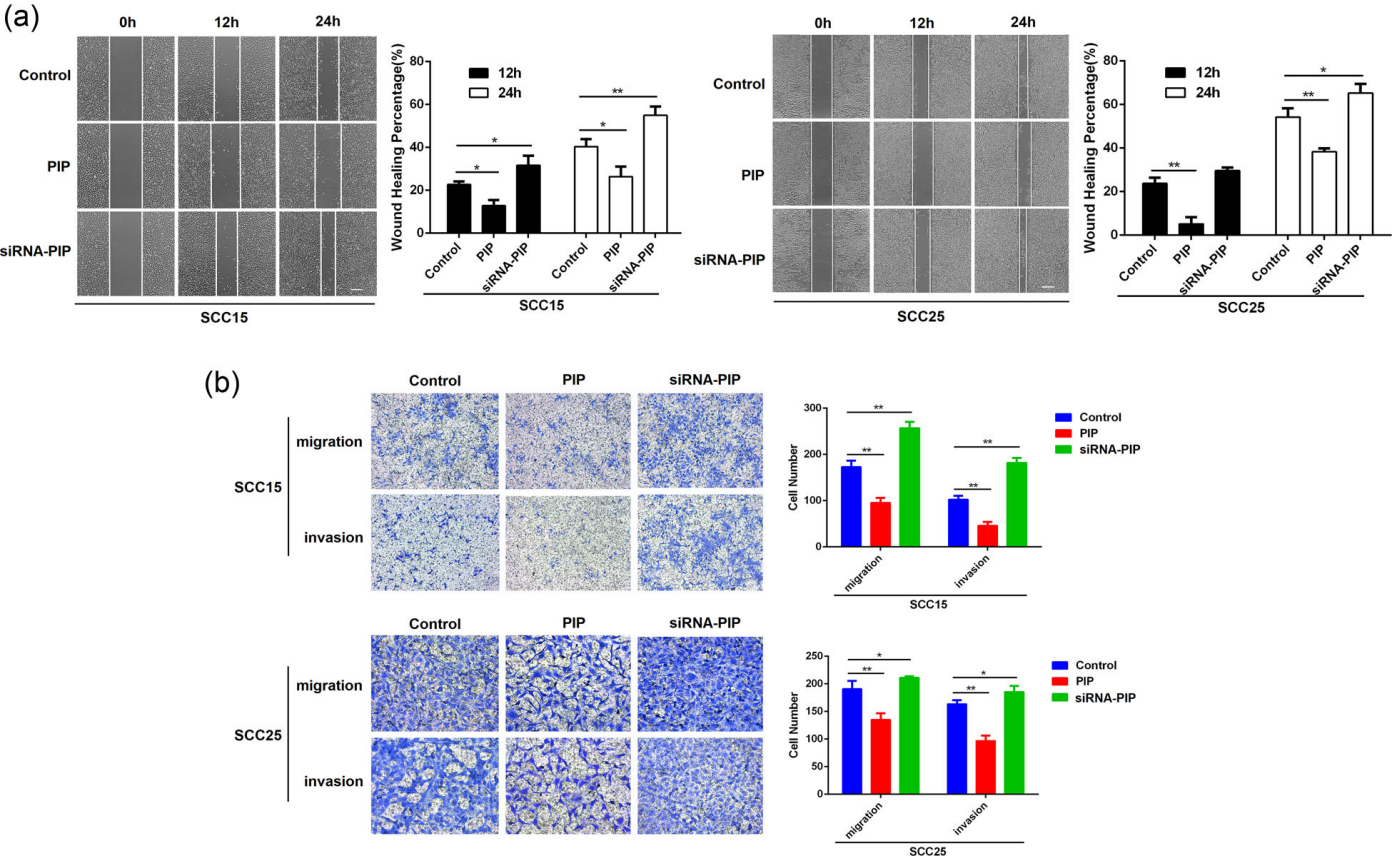
The effects of PIP on OSCC migration and invasion in vitro. (a) The view of wound‐healing migration assay; the average rate of SCC15 and SCC25 cells migration at 12 and 24 hr after control, PIP vector or siRNA‐PIP transfection. All data were shown as mean ± *SD*. *p < 0.05, **p < 0.01. (b) The results of the transwell assay of SCC15 and SCC25 cells at 24 hr after control, PIP vector or siRNA‐PIP transfection; relative ratios of migrated and invasive cells per field are shown. All data were shown as mean ± *SD*. *p < 0.05, **p < 0.01. OSCC: oral squamous cell carcinoma; PIP: prolactin‐inducible protein; *SD*: standard deviation; siRNA: small interfering RNA [Color figure can be viewed at wileyonlinelibrary.com]

As shown in Figure 3a, PIP overexpression significantly inhibited the migration of SCC15 and SCC25 cells in wound‐healing assays as evidenced by the narrowed healing borders. PIP silencing produced the opposite effect (*p* < 0.05). To confirm these findings, transwell assays were performed. Migration assays revealed that the number of PIP overexpressed SCC15 and SCC25 cells crossing the membrane were significantly reduced significantly compared with control cells (*p* < 0.01). However, PIP silencing in SCC15 and SCC25 cells significantly increased the number of cells crossing the membrane border (*p* < 0.05). Invasion assays produced similar findings (*p* < 0.05; Figure 3b). Taken together, these data suggest that the overexpression of PIP inhibits the migration and invasion abilities of SCC15 and SCC25 cells, whilst PIP silencing produces the opposite effect.

### 3.4 IHC analysis of PIP expression in OSCC specimens

**Figure 4:**
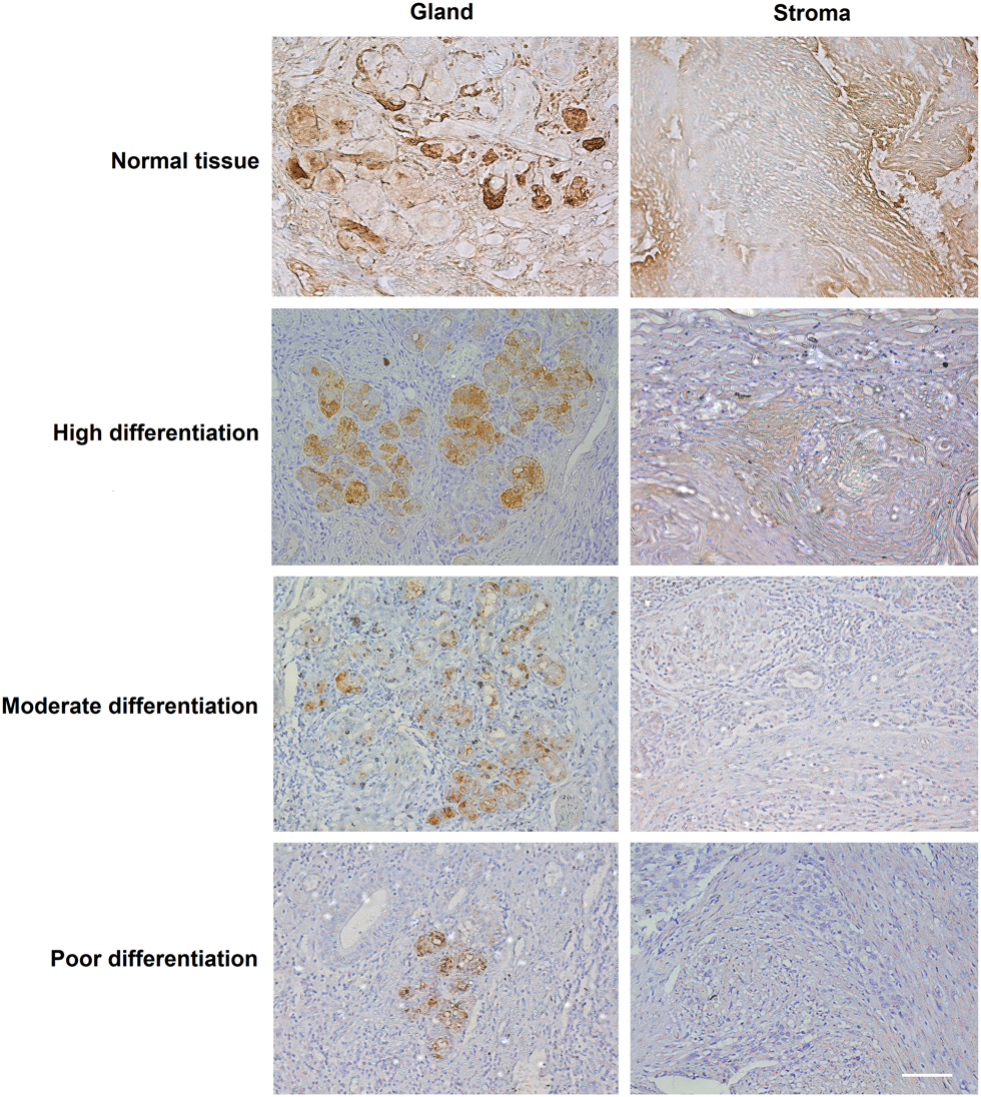
PIP expression in OSCC tissues. Immunohistochemistry (IHC) analysis of PIP in OSCC and adjacent normal tissues (*n* = 45; ×20). OSCC: oral squamous cell carcinoma; PIP: prolactin‐inducible protein [Color figure can be viewed at wileyonlinelibrary.com]

We divided the 45 cases into four groups according to tissue type. From IHC staining, we observed PIP expression in both gland and stromal cells. The expression levels of PIP were high in adjacent nontumor and highly differentiated tissues, compared with moderately and poorly differentiated tissues (Figure 4).

### 3.5 PIP influences cell signaling

**Figure 5:**
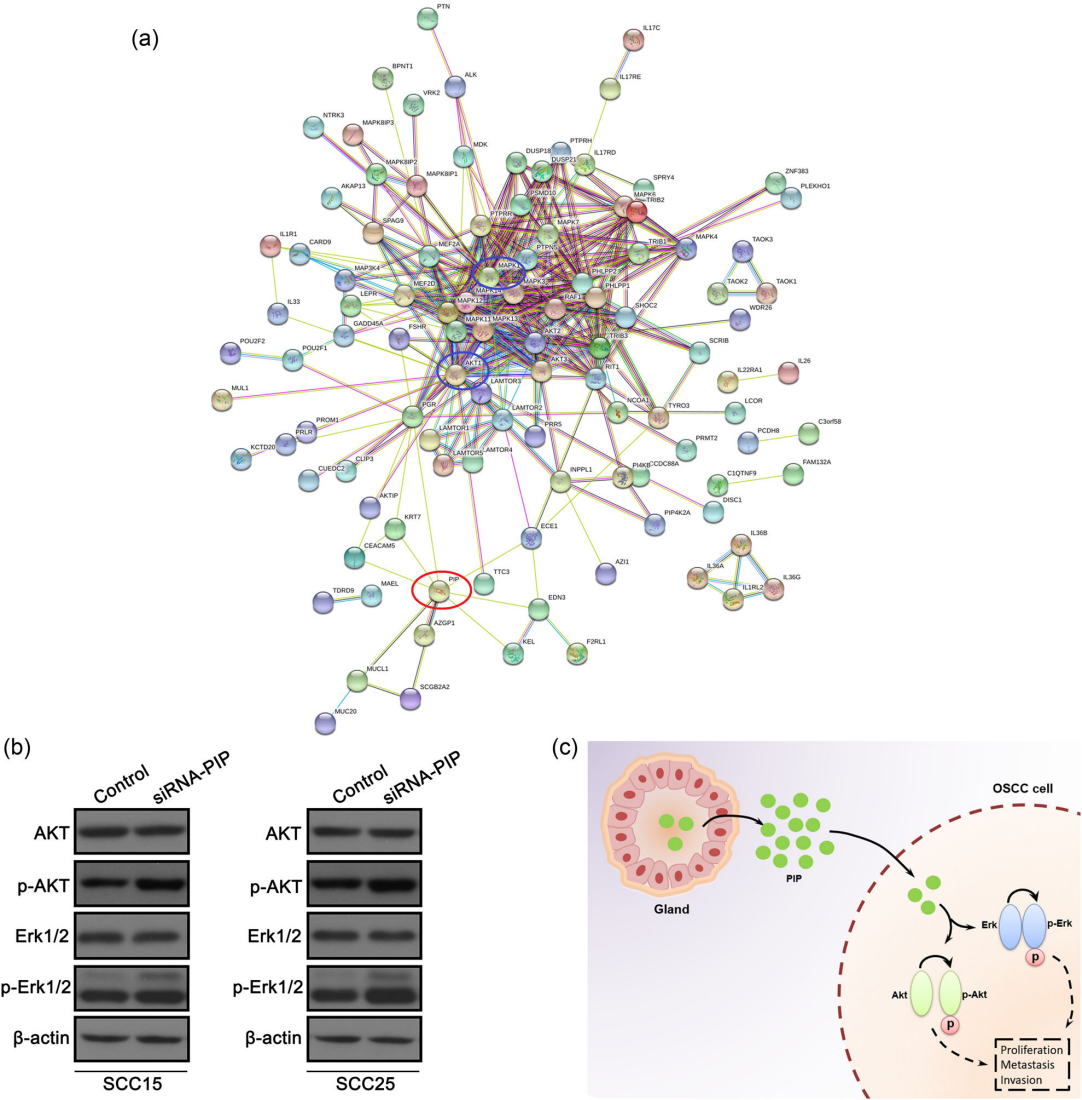
PPI network suggests that PIP interacts with numerous components of the AKT/MAPK pathway. (a) PPI network for PIP was retrieved from the STRING database and reconstructed by adding recently identified PIP interacting proteins. (b) Western blot analyses in SCC15 and SCC25 cells after transfection with control, PIP vector, and siRNA‐PIP. β‐Actin served as an internal control. (c) Proposed model for the effects of PIP on OSCC cell proliferation, invasion, migration and AKT/MAPK signaling. AKT/MAPK: Akt/mitogen‐activated protein kinase; OSCC: oral squamous cell carcinoma; PIP: prolactin‐inducible protein; PPI: protein–protein interaction; siRNA: small interfering RNA [Color figure can be viewed at wileyonlinelibrary.com]

PPI network analysis suggested that PIP was associated with components of the AKT/MAPK signaling axis. We therefore assessed the expression levels of AKT and ERK1/2 (Figure 5a). The expression levels of p‐ERK1/2 and p‐AKT were significantly higher in siRNA‐PIP cells compared with controls. No changes in total ERK and AKT expression were observed confirming a specific effect on kinase activity (Figure 5b). Based on the evidence that PIP was is a secretory protein (Parris et al., 2014), our results suggest that PIP is likely to be secreted by glandular cells to inhibit OSCC cells proliferation, migration, and invasion (Figure 5c).

### 3.6 PIP inhibits the proliferation, migration, and invasion of OSCC through AKT/MAPK signaling pathways

**Figure 6:**
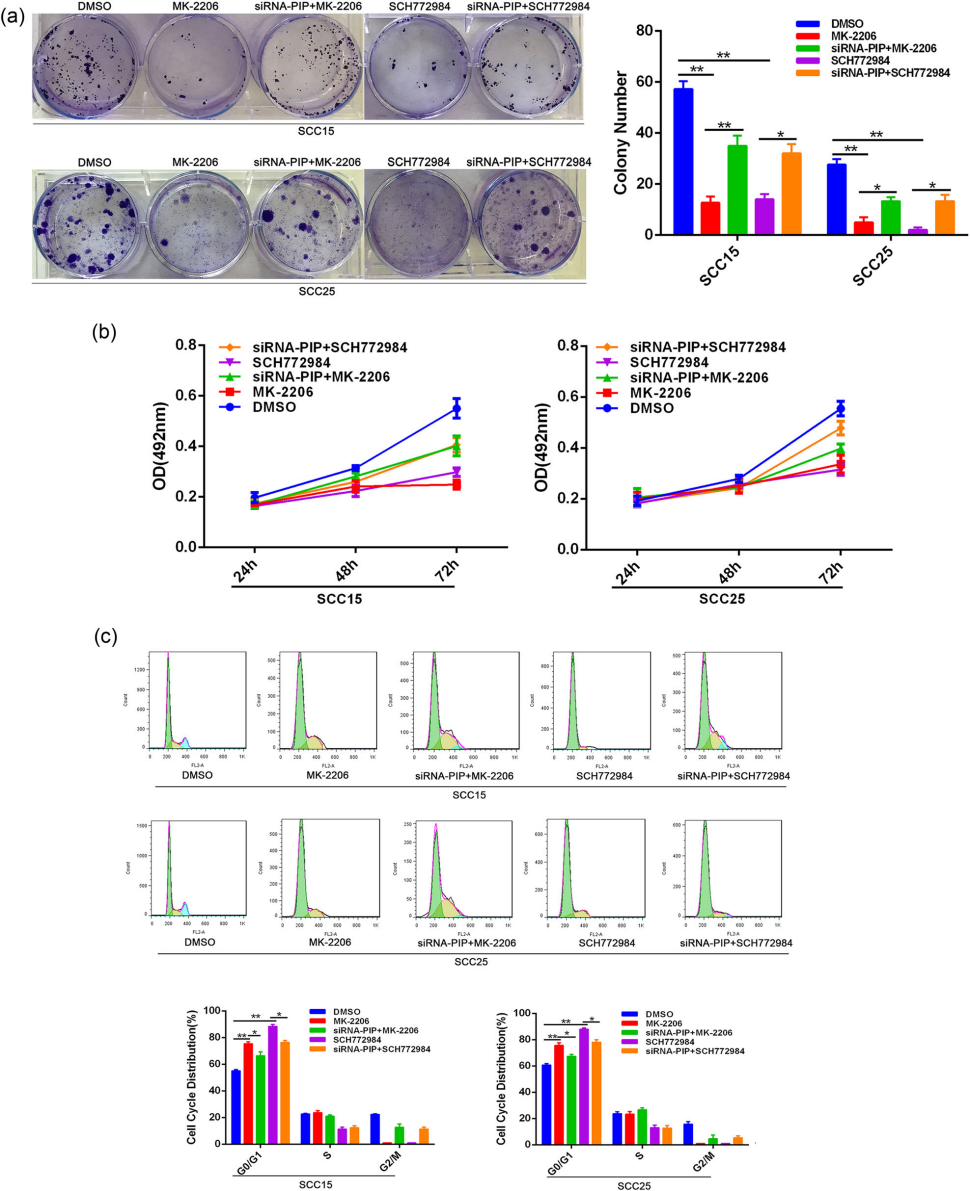
Effects of siRNA‐PIP coculture and AKT/MAPK inhibitor on OSCC cell proliferation. (a) Cells treated with DMSO, MK‐2206, SCH772984, siRNA‐PIP + MK‐2206 or siRNA‐PIP + SCH772984 were assessed by colony forming assays as described in Figure 2(a). All data were shown as mean ± *SD*. *p < 0.05, **p < 0.01. (b) At 24, 48, and 72 hr posttreatment with DMSO, MK‐2206, SCH772984, siRNA‐PIP + MK‐2206 or siRNA‐PIP + SCH772984, cell proliferation was examined via MTT assays. (c) Cell cycle analysis of SCC15 and SCC25 cell cycle treated as in (a). All data were shown as mean ± *SD*. *p < 0.05, **p < 0.01. AKT/MAPK: Akt/mitogen‐activated protein kinase; DMSO: dimethyl sulfoxide; MTT: 3‐(4,5‐dimethyl‐2‐thiazolyl)‐2,5‐diphenyl‐2H‐tetrazolium bromide; OSCC: oral squamous cell carcinoma; PIP: prolactin‐inducible protein; *SD*: standard deviation; siRNA: small interfering RNA [Color figure can be viewed at wileyonlinelibrary.com]

**Figure 7:**
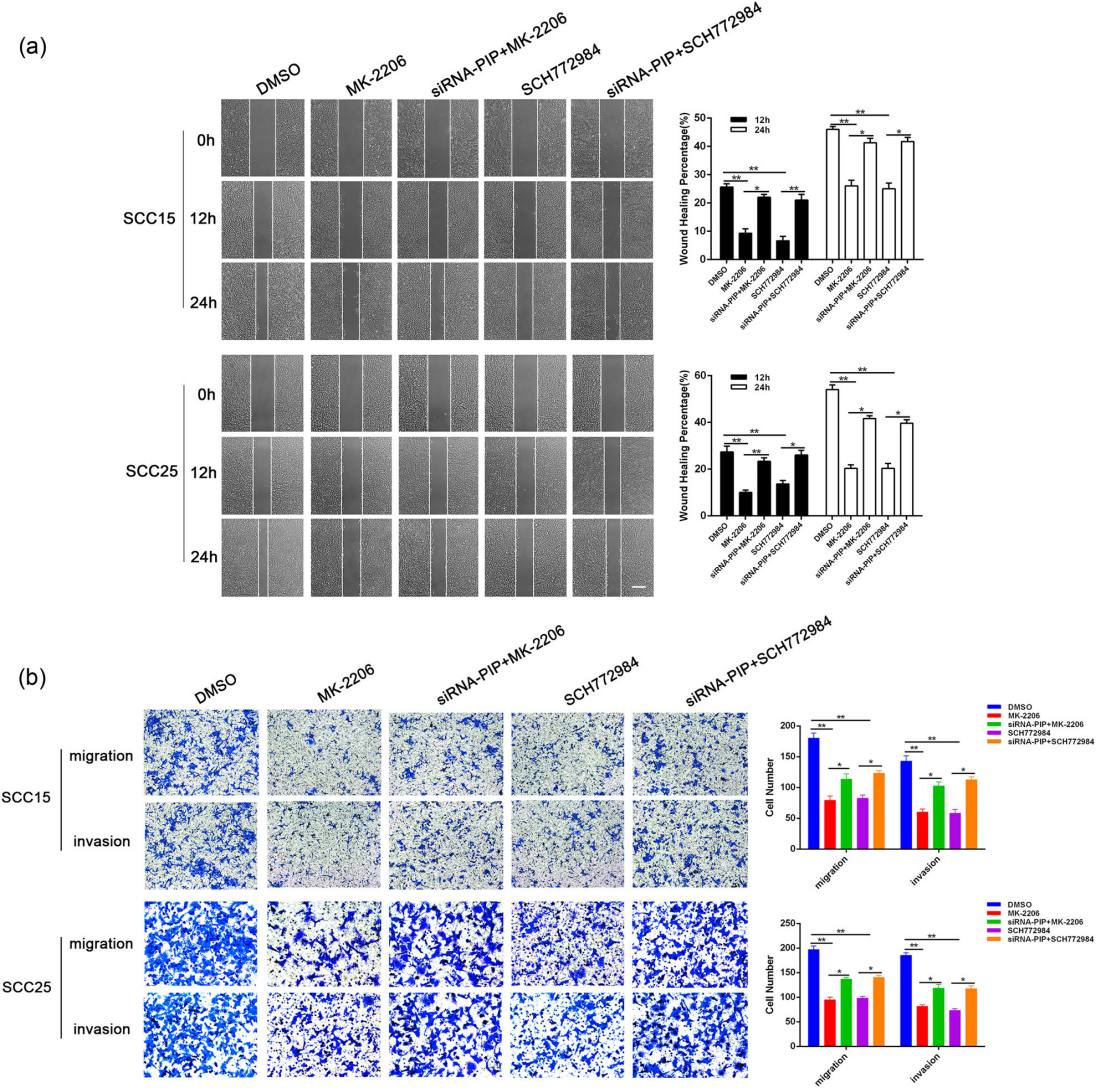
Effects of siRNA‐PIP coculture and AKT/MAPK inhibitor on OSCC migration and invasion. (a) Wound‐healing migration assays; average rates of SCC15 and SCC25 cells migration at 12 and 24 hr after treatment as in Figure 6(a). All data were shown as mean ± *SD*. *p < 0.05, **p < 0.01. (b) Transwell assays of cells treated as in (a); relative ratio of migrated and invasive cells per field is shown. All data were shown as mean ± *SD*. *p < 0.05, **p < 0.01. AKT/MAPK: Akt/mitogen‐activated protein kinase; OSCC: oral squamous cell carcinoma; *SD*: standard deviation; siRNA: small interfering RNA [Color figure can be viewed at wileyonlinelibrary.com]

To investigate the effects of AKT/MAPK signaling on OSCC growth, we assessed the effects of the AKT inhibitor MK‐2206 and ERK inhibitor SCH772984. Clonogenic and MTT experiments showed that MK‐2206 and SCH772984 could inhibit colony formation and reduced proliferation. When MK‐2206 and SCH772984 were added to siRNA‐PIP cocultured cells, their inhibitory effects diminished (Figure 6a,b). Cell cycle analysis revealed that MK‐2206 and SCH772984 induced G0/G1 OSCC cells which could be rescued by PIP silencing (*p* < 0.05; Figure 6c). Figure 7a shows that MK‐2206 and SCH772984 treatment also inhibited the migration of SCC15 and SCC25 cells which could be recovered by siRNA‐PIP Coculture (*p* < 0.05). Furthermore, transwell migration assays indicated that the number of both SCC15 and SCC25 cells crossing the membrane following MK‐2206 or SCH772984 treatment significantly decreased in comparison to solvent treated cells (*p* < 0.01). Coculture with siRNA‐PIP cells increased the number of cells passing through the membrane crossing the membrane following drug treatment (*p* < 0.05). Invasion assays followed an identical pattern (Figure 7b). Taken together, these data demonstrate that PIP silencing can compensate for the inhibitory effects of MK‐2206 and SCH772984 on SCC15 and SCC25 migration and invasion.

## 4 DISCUSSION

OSCC is the most malignant subtype of squamous carcinoma in the head and neck. Although a panel of molecular biomarkers, including p53, Retinoblastoma, and p16, have been reported as indicators for OSCC prognosis (Feng, Xu, & Chen, 2012), none can dictate clinical decision‐making processes due to their involvement in complex signaling pathways and multiple uncharacterized interactions (Zhu et al., 2014). In this study, using iTRAQ combined with 2D LC‐MS/MS, we comprehensively analyzed DEPs with molecular functions related to OSCC and performed a comparison to paired normal tissue. The iTRAQ data were verified by western blot analysis and cell culture assays.

A recent study discovered that PIP expression reduced gradually along with higher stage and grade of breast cancer (Urbaniak, Jablonska, Podhorska‐Okolow, Ugorski, & Dziegiel, 2018). Consistently, according to our results, they were shown that low levels of PIP expression are associated with a worse grade. The finding (Vanneste & Naderi, 2015) that PIP silencing led to a marked reduction in cell adhesion allowed us to interpret PIP silencing could promote migration and invasion in OSCC cells. In addition, Gangadharan et al. (2018) similarly observed considerable downregulation of PIP transcription in cancer samples compared with normal breast tissue. We also found PIP expression in OSCC tissue is lower than normal tissue. Moreover, the network in a previous study (Debily et al., 2009) identified appears connected with an inhibition of proliferation coupled with an increase of apoptosis in breast cancer cell lines which aid us to explain our results of clonogenic and MTT assays.

PIP is a secretory glycoprotein found primarily in apocrine and mucosal tissues, including breast tissue and the salivary glands (Blanchard et al., 2009; Parris et al., 2014; Priyadarsini et al., 2014). PIP binds to a diverse range of oral bacteria, signifying its protective function in the oral mucosa through inhibiting bacteria settlement and growth (Nistor, Bowden, Blanchard, & Myal, 2009). PIP has also been shown to belong to a group of salivary proteins, whose profusion is reduced in the presence of oral bleeding (Fleissig et al., 2010; Haigh et al., 2010). PIP has also been reported to associate with secretory lesions and is related to atypical and benign precursors (Asirvatham, Falcone, & Kleer, 2016; Gangadharan et al., 2018; Gown, Fulton, & Kandalaft, 2016). In our study, PIP was found to regulate cell proliferation, migration, and invasion of OSCC cells. We believe that these effects are due to the presence of a large number of salivary glands in the oral environment that can secrete PIP in the presence of OSCC, allowing it to enter the stroma and act on OSCC cells for further regulation.

Although these results are promising, this study has some limitations. We did not validate our findings in vivo which may affect the reliability of the data. According to our previous studies, a correlation between PIP and cell adhesion requires investigation. Simultaneously, experiments exploring the mechanism of PIP mediated regulation of OSCC cell growth requires further studies. We did however identify that PIP expression is significantly lower in both OSCC cells and tissue. These findings may aid clinical diagnosis should PIP expression be related to the degree of OSCC malignancy. PIP may also benefit the early diagnosis of OSCC, and therefore improve both treatment and patient prognosis.

In summary, iTRAQ analyses followed by high‐throughput 2D LC‐MS/MS were used to screen DEPs in OSCC and normal tissue in patient samples for the first time. We provide a comprehensive insight into PIP and its use as a novel biomarker of OSCC. We believe that these findings can improve clinical efficacy and reduce OSCC related mortality in future studies.

## CONFLICTS OF INTEREST

The authors declare that there are no conflicts of interest.

## AUTHOR CONTRIBUTIONS

L. G. and K. Z. conceived and designed the experiments; Q. W., Y. Z., and Y. W. performed the experiments; S. L., Z. D., X. X. and X. Q. analyzed the data; Q. W. and W. R. wrote the paper; C. J, X. L., and K. Z. participated in the data analysis and the paper revision.
